# Points in Practice1Read before the Missouri State Veterinary Association, Moberly, Sept. 4, 1895.

**Published:** 1895-10

**Authors:** Junius H. Wattles


### POINTS IN PRACTICE.[1]

BY JUNIUS H. WATTLES, D.V.S.

I HAVE selected for my subject "Points in Practice" for several reasons, and not the least is the belief that it will bring out a discussion of the several subjects touched upon, and the hope that we will all become gainers by absorbing the ideas of our fellow-practitioners.

It is often said that a lazy man takes the most pains to devise easy methods, and you will please accept my acknowledgment now that my desire to avoid anything like labor has become chronic, and that the hardest labor performed by me is that of finding easy ways of doing things. This will explain to you why my subject is a practical rather than a theoretical one, as to-day the theorist is the hardest worker in our ranks.

The wisdom of the world has been gained by close observation of minute things, and some of the most practical points are sometimes obtained from the humblest sources.

In our practice we are frequently called upon to perform the very common-place operation of castrating a "tom-cat," and from a student attending my lectures came the welcome intelligence to me of an easy manner of confinement during the operation.

Follow instructions closely: Take the right posterior limb of the animal between the third and fourth fingers of the right hand; the right anterior limb between the first and second fingers of the right hand; take the right ear between the thumb and first finger of the right hand. Use the left hand in a similar manner on the left side of the animal. Your assistant, holding him firmly, will bring the animal back toward the assistant and facing the operator, in which position the operation may be performed with "neatness and dispatch," aided by the proper application of a sharp knife.

Men to-day do not accept new theories as positive facts until they are proven, and if in our practice an error is discovered in the methods of treatment that our text-books and preceptors have advocated, we are not doing ourselves justice unless we

[1] Read before the Missouri State Veterinary Association, Moberly, Sept. 4, 1895.

make an effort to rectify that error and to try and find a more satisfactory way for ourselves and patrons.

It is a very common occurrence in our practice to be called upon to treat open joints in horses, and we can all justly consider these cases as stumbling-blocks in practice, as the majority of us would probably testify to very unsatisfactory results obtained in the greater number of cases; and in hopes that some benefit may be derived from misfortune, this point has been selected for to-day.

When we first enter practice in this profession, we usually follow the lines laid down by our predecessors when called to treat one of these cases, and we use hot water and cold water, poultices and pounded ice, liniments and injections, with the unsatisfied feeling that if by any chance the poor animal lives that it would be more than likely to have complete anchylosis of the affected joint. This in itself was sufficient inducement for me to cast about for something that would give better results, and the following are my conclusions:

Make it a positive rule never to inject anything into an open joint unless there is a discharge of pus from the wound, and then nothing but some bland, non-irritating substance, such as olive oil or lard oil. These can be used with an unsparing hand for the first treatment to mechanically remove the accummulation of pus, and is not to be repeated unless absolutely necessary. When one is called before the care-taker has applied or injected someone of the usual liniments, there will be no reason for injecting anything into the wound.

It is necessary in all cases to provide a large pad of absorbent cotton, a long roller bandage, either cotton or flannel; also a plentiful supply of oxide of zinc, seven parts; powdered wood charcoal, one part. First apply to the wound a part of the powder, then cover the pad of cotton with a liberal quantity of the same; having the pad held against the wound by an assistant, apply the bandage rather snugly and give positive orders to let it alone for twenty-four hours, when the bandage may be removed carefully without wetting it and reapplied. You will observe that the most important part is to keep all wet applications away from the wound, and not to meddle with the dressing as long as the discharge does not wet through the bandage. In case of offensive odor, a small quantity of powdered permanganate of potash may be added to the powder.

It seems to me that the use of the trocar and canula in gastric flatulence is entirely uncalled for, and many of us will be obliged to confess our inability to use such an instrument. The necessity for using such an instrument, or rather the condition for which it would be used, can be met by a piece of rubber-tubing one-half inch in diameter and eight feet long. Stand facing the animal; wet the tubing, pass it into the left nostril of the animal, keeping it against the septum nasi until the pharynx is reached; then against the roof of the pharynx, and it easily passes down the œsophagus into the stomach, allowing the gas to escape, and furnishing the means to pass medicine directly into the stomach, if desired.

In cases of obstruction of any sort in the œsophagus the same plan can be followed in passing the tubing. In obstruction from dry material, as oats, bran, or chopped feed of any sort, pass the tube down to the obstruction, and then pour large quantities of water or oil down the tube; this will float the dry material up, and it is either passed out through the tube or through the opposite nostril. In cases of obstruction from such articles as potatoes, apples, or turnips, pour down the tube either olive oil or linseed oil, containing a full dose of chloroform. The oil lubricates the substance and the parts, and the anæsthetic relaxes the muscle-fibres, allowing the obstacle to pass down into the stomach.

After the operation of œsophagotomy food may be passed into the stomach until such time as the wound will permit the animal to take solid food, by using the same method.

It is sometimes necessary to anæsthetize our equine patients when performing operations, and one of the simplest and best arrangements that can be made for the purpose consists of a tube or cylinder of cloth, about six inches in diameter and fourteen inches long, open at both ends and provided with a puckering string at each end. One end of the tube is passed over the nostrils, being carried into the mouth far enough so that the upper string will be above the canine teeth in the male. The other end of the tube contains the sponge, saturated with the anæsthetic, and is closed by the lower string, which can be loosened as required. This simple arrangement allows sufficient air to pass into the lungs through the mouth in cases where chloroform is used, and a horse can be kept under its influence for a very long time with very little danger to the patient.

Not only in city practice, but in country practice as well, are we called upon to treat wounds of the feet, usually produced by nail punctures, and supplemented by the addition of some caustic agent to the wound by some well-meaning person. It seems to me that the use of irritating applications in punctured wounds of the feet is not only uncalled for, but is positively dangerous in the majority of cases, as they cauterize the external opening of the wound and imprison the pus, that is almost sure to form if the opening is closed by irritants, and compel the pus to seek an outlet through the soft tissues, usually at the heel. My advice to my patron is: When a horse picks up a nail stop at once and remove the nail; have the horseshoer enlarge the opening in the sole; have the foot immersed for fifteen minutes in hot water if in winter, and if in summer for an hour in cold water. My reason for using hot water in winter and cold in summer is because we can add comfort to the animal by so doing, and not from any theoretical idea. Cases treated in this manner have never, to my knowledge, reached the suppurative stage.

In cases where suppuration is established when called, my practice is to open the abscess at the coronet, unless it is already open, which is usually the case, and then enlarge the opening in the sole, as it is absolutely necessary to have free drainage to facilitate recovery. Formerly, like many others, my practice was to poultice; but this method of treatment, with many others, has been relegated to the "has been" corner, after it had added its quota of gray hair to my already bountiful crop, that soon followed my poulticing in an outbreak of furuncle some years ago.

If you have not already satisfied yourselves on the subject, try my method of treating the suppurating diseases of the feet by injections of linseed oil, carbolic acid, and where there is pain in the parts, the addition of chloroform.

Two years ago, at our meeting of this Association in Clinton, my subject was "Fistulous Withers," and my theory at that time was that it was a specific contagious disease, and the same opinion still exists in my mind. But leaving out the theoretical points, and considering nothing but the practical phases, it seems to me that after we had seen this very common disorder treated for a lifetime by caustics and the knife with the poorest sort of success, that we are justified in discarding as far as possible the irrational and barbarous methods, and casting about for a new one.

My observation convinces me that fistula is permanently cured by mild methods of treatment and is aggravated by severe methods, and that a larger percentage of cases are cured by the very mildest applications than are cured in any other way.

With this idea in mind, my plan is to use unlimited applications of cold water, and when the abscess has not pointed, or the formation of pus is limited, to use iodine in some one of its forms externally, and hyposulphite of soda internally. In the cases where there is an external opening the cold-water applications are the same, and the injections into the abscess are such as will produce the very least irritation, and are used after thoroughly flushing out the cavity with clean cold water. My belief is that fistulous withers and malignant carbuncle of man are first cousins.

Another subject, and my effort to interest you is finished. My paper on the subject of "Hypodermic Injections in the Treatment of Ossific Diseases of the Joints" has brought to me many letters, and my suggestion to those who have not already made a trial of such treatment is to do so, and make some sort of a report at some of our meetings, as it is by an interchange of ideas that we are able to progress toward the highest point of perfection in our truly honorable, scientific, and humane profession.

